# Prognostic Impact of Anterior Commissure Involvement on Treatment Failure in Early‐Stage Glottic Carcinoma Treated With Definitive Radiotherapy: A Univariate and Multivariate Meta‐Analysis

**DOI:** 10.1002/hed.70219

**Published:** 2026-03-02

**Authors:** Francesca De Felice, Luca Passalacqua, Giuseppe Sanguineti

**Affiliations:** ^1^ Radiation Oncology, AOU Policlinico Umberto I, Department of Radiological, Oncological and Pathological Sciences “Sapienza” University of Rome Rome Italy; ^2^ Department of Statistical Sciences “Sapienza” University of Rome Rome Italy; ^3^ Radiation Oncology Unit IRCCS Regina Elena National Cancer Institute Rome Italy

**Keywords:** anterior commissure, glottic cancer, local recurrence, prognosis, radiotherapy

## Abstract

**Background:**

Early‐stage glottic carcinoma is characterized by high cure rate, but about 10% of patients still develop local recurrence after radiotherapy (RT). Although advances in local therapies have markedly improved larynx preservation, anterior commissure involvement (ACI) may worsen outcomes yet remains insufficient to fully guide personalized treatment. Here, we conducted a univariate meta‐analysis and a Bayesian multivariate meta‐analysis of multiple factors (MVMA‐MF) to evaluate the prognostic impact of ACI and on local failure in early‐stage glottic carcinoma.

**Methods:**

We conducted a systematic review with univariate meta‐analysis and MVMA‐MF using data from studies of early‐stage glottic carcinoma treated with definitive RT, incorporating clinical information, risk factors and survival outcomes. A systematic literature search was conducted in PubMed and Scopus on Sep 27, 2025. Eligible studies provided sufficient data to estimate odds ratios (ORs) for local control in relation to patient, tumor and treatment factors, including ACI. Data extraction followed PRISMA guidelines, and study quality was assessed using MINORS criteria. The primary endpoint of the study was the effect of ACI on local failure. Pooled ORs were calculated with a random‐effects model, and heterogeneity was assessed using the Cochrane *Q* test and *I* ^2^ statistic. To address correlations among risk factors, we applied a Bayesian multivariate meta‐analysis of multiple factors (MVMA‐MF), testing three models (H, M0, U) with Markov Chain Monte Carlo algorithm.

**Findings:**

Nine clinical trials representing 2527 patients were included. In univariate meta‐analysis, the presence of ACI was significantly associated with an increased risk of local recurrence in early‐stage glottic carcinoma treated with RT (OR 1.61, 95% CI 1.15–2.26, *p* = 0.006), with moderate heterogeneity (*I* ^2^ = 34%). In MVMA‐MF including 14 risk factors, only T substage demonstrated a consistent and statistically significant association with recurrence across all models. ACI, smoking status and other risk factors showed non‐significant associations, with wide credible intervals overlapping unity. Correlation analysis revealed mostly low‐to‐moderate inter‐variable correlations, supporting the multivariable modeling approach. Comparison of univariate and multivariate estimates for ACI indicated that the unadjusted analysis suggested increased recurrence risk, whereas adjusted models yielded attenuated and non‐significant associations (adjusted ORs 1.05–1.43, all 95% CrIs including unity).

**Interpretation:**

This study represents the first application of both univariate and multivariate meta‐analysis to clarify the prognostic role of ACI in early‐stage glottic carcinoma treated with definitive RT. While univariate analysis suggested ACI increased recurrence risk, this effect was not confirmed in MVMA‐MF, indicating the univariate findings may be confounded by correlation with other factors, particularly T substage. MVMA‐MF approach accounted for heterogeneity, collinearity and incomplete reporting, providing more robust evidence. This work underscores the value of advanced multivariate methods in oncology meta‐analysis and provides the strongest evidence to date that ACI should not be regarded as a fundamental prognostic criterion in early‐stage glottic carcinoma.

## 1 Introduction

Current treatment modalities for early‐stage glottic carcinoma aim at larynx preservation and include conservative surgery—either via transoral laser or open technique—or radical radiotherapy (RT) [1]. The presence of anterior commissure involvement (ACI) can negatively impact both oncological and functional outcomes, regardless of the therapeutic approach used [1, 2]. Despite the high cure rate with a 5‐year overall survival (OS) of 79% and 5‐year disease specific survival of 96%, approximately 10% of patients with early‐stage glottic carcinoma experience local failure after definitive RT [2]. In these cases, salvage surgery with partial/total laryngectomy—based on recurrence stage—is often necessary [1]. Understanding whether ACI independently contributes to treatment failure—although ACI does not change TNM stage, it is used to upgrade patients to a high‐risk cases due to the anatomical complexity—would be valuable in selecting patients who might benefit from upfront laryngeal surgery to improve local control.

In this context, we conducted a univariate meta‐analysis and a Bayesian multivariate meta‐analysis of multiple factors (MVMA‐MF) to comprehensively assess the prognostic impact of ACI on local recurrence in early‐stage glottic carcinoma treated with RT. By modeling the correlations among clinical risk factors, this approach aims to support more individualized risk stratification and guide treatment selection.

## 2 Methods

A structured literature search of the PubMed and Scopus databases were searched for “radiotherapy” or “radiation therapy,” “early glottic” or “early larynx” or “early laryngeal,” “cancer” or “carcinoma” in title and abstract. Only clinical trials, written in English, were considered. Included studies were cohort analysis or case–control studies of early‐stage glottic carcinoma patients treated with definitive RT with sufficient data to evaluate the log odds ratios (ORs) and their 95% credible intervals (CrIs). Reference lists of previously published reviews were explored. The Preferred Reporting Items for Systematic Reviews and Meta‐Analyses (PRISMA) statement was followed to perform the meta‐analysis [3]. The last search was carried out on 27 September 2025. In the closer evaluation of potentially eligible articles, when two articles appeared to report results with overlapping data, only the data representing the most recent publication date or with the larger sample size were included in the meta‐analysis. We made every attempt to eliminate redundancy in the data represented in our meta‐analysis. From all included studies, the following data were extracted: first author' surname, publication year, sample size of cases, treatment characteristics, OR and 95% confidence interval (CI) from the univariate analysis assessing the correlation between local control and various patient and tumor characteristics, as well as clinical outcomes. When relevant data were not reported, corresponding authors were contacted to request the missing information. The primary endpoint was to estimate the effect of ACI on local failure. The recorded risk factors included: ACI, T substage, vocal cord mobility, subglottic extension, tumor size, beam energy, RT technique, total RT dose, fraction size, overall treatment time (OTT), field size, waiting time, gender, arytenoid protection, hemoglobin (Hb) level, smoking status, age, tumor grading, surgical fitness, pre‐existing laryngitis, and alcohol abuse. Data were estimated during the median follow‐up of each trial.

Study quality was quantified using the methodological index for nonrandomized studies (MINORS) criteria [4]. Statistical analysis was performed using Review Manager (RevMan) version 5.3 (http://www.cochrane.org) and RStudio version 0.98.1091.

Individual and pooled ORs with 95% confidence intervals (CI) were calculated using a random‐effects model. A two‐sided *p*‐value < 0.05 was considered statistically significant. Statistical heterogeneity among studies was assessed using the Cochrane *Q* test (significant at *p* < 0.10) and the *I* ^2^ value (indicating substantial heterogeneity if > 50%) [5]. The main limitation of this analysis is the use of binary outcomes (presence or absence of local recurrence) without accounting for time‐to‐event data (time to recurrence, duration of follow‐up or censoring). As a result, the analysis was based on ORs rather than hazard ratios limiting the ability to evaluate the temporal dynamics of recurrence risk. These factors, along with heterogeneity across studies may introduce bias and limit the interpretability of the pooled effect estimate.

A Bayesian MVMA‐MF model was also used because of the sparsity of data [6]. Univariate models may yield biased or imprecise estimates of overall effect sizes, especially when several factors (such as ACI and T substage) are expected to be correlated. This suggests that a multivariate model may be more appropriate for the analysis. Considering our primary endpoint, the multivariate approach allows for an adjusted estimation of the effect of ACI by accounting for the joint distribution and potential correlations among the other covariates. This results in more robust and reliable effect size estimates compared to those obtained from separate univariate analysis. Because within‐study correlations were unknown, we applied three models: Model H (accounting for both within‐ and between‐study correlations), Model M0 (accounting for between‐study correlations) and Model U (a univariate model ignoring all correlations) to estimate the overall log ORs of the risk factors. These included: (i) ACI (yes vs. no); (ii) T substage (T1a vs. T1b/T1 vs. T2); (iii) vocal cord mobility (impaired vs. not impaired); (iv) subglottic extension (yes vs. no); (v) RT technique; (vi) total RT dose (<60 Gy vs. ≥ 60 Gy); (vii) fraction size (conventional vs. accelerated); (viii) OTT (≤ 40 days vs. >40 days); (ix) field size (T vs. TN); (x) gender (male vs. female); (xi) Hb level (normal vs. low); (xii) smoking status (yes vs. no); (xiii) age (< 65 years vs. ≥ 65 years); (xiv) tumor grading (low vs. intermediate/high). Log ORs derived from adjusted multivariate regression models, were excluded to maintain consistency across studies. Given the sparse nature of the dataset, all models were implemented using Bayesian methods. The posterior estimates were based on three Markov Chain Monte Carlo (MCMC) chains, each containing a run of 100 000 updates after a 100 000‐run burn‐in period. The convergence of the chains was checked using their trace plots.

Finally, ACI multivariate log ORs were exponentiated to yield adjusted ORs for comparison with univariate ORs.

## 3 Results

### 3.1 Included Studies

**FIGURE 1:**
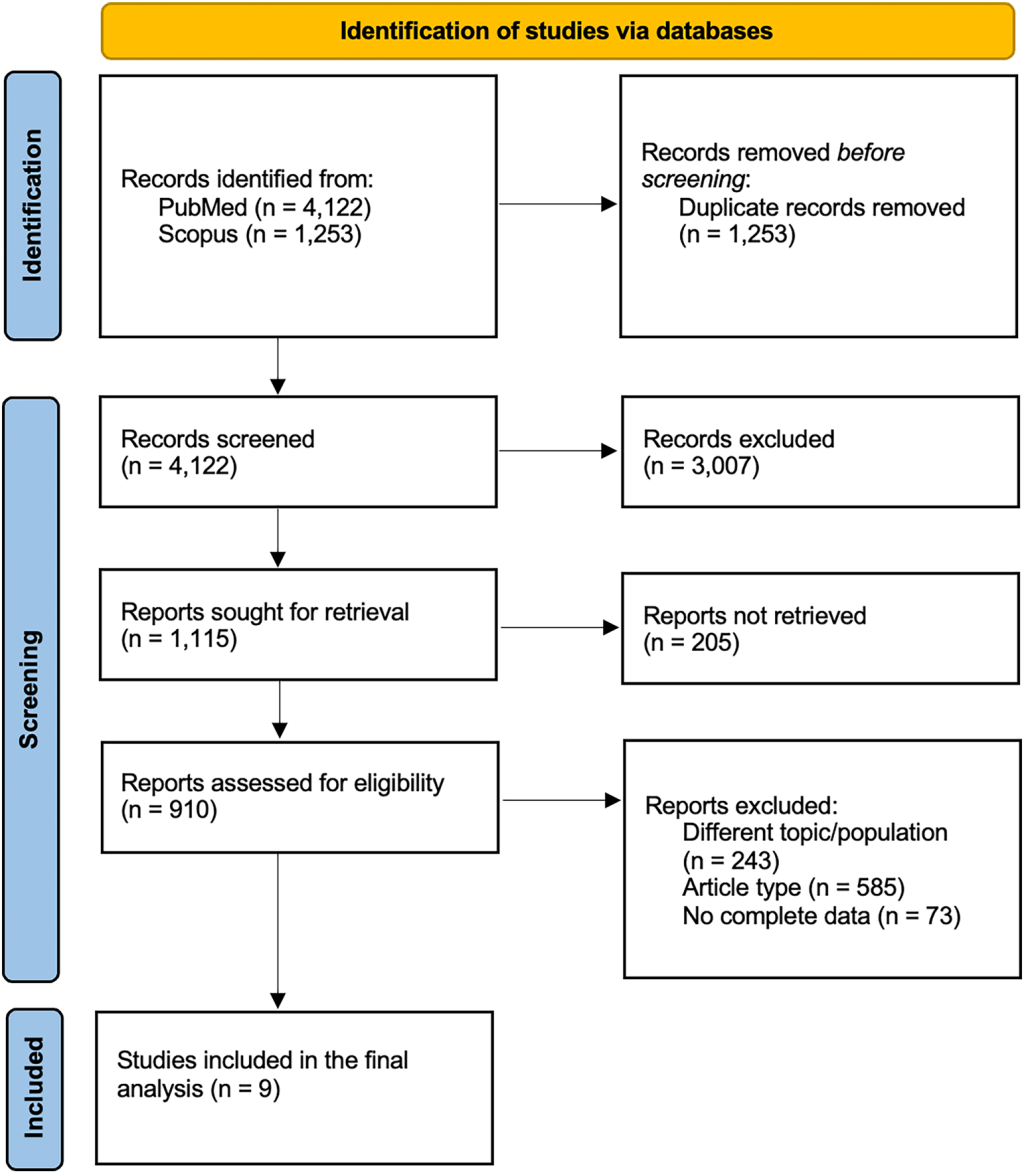
PRISMA flow chart. [Color figure can be viewed at wileyonlinelibrary.com]

The meta‐analysis included nine clinical trials (2527 patients) [7, 8, 9, 10, 11, 12, 13, 14, 15]. Flowchart of the retrieved studies is presented in Figure 1. Included studies were published between 2000 and 2023. All but one were retrospective in design, with median follow‐up periods ranging from 33 months to 9.8 years. The median study quality index was 16 and no significant evidence of publication bias was detected.

### 3.2 Univariate Meta‐Analysis

**FIGURE 2:**
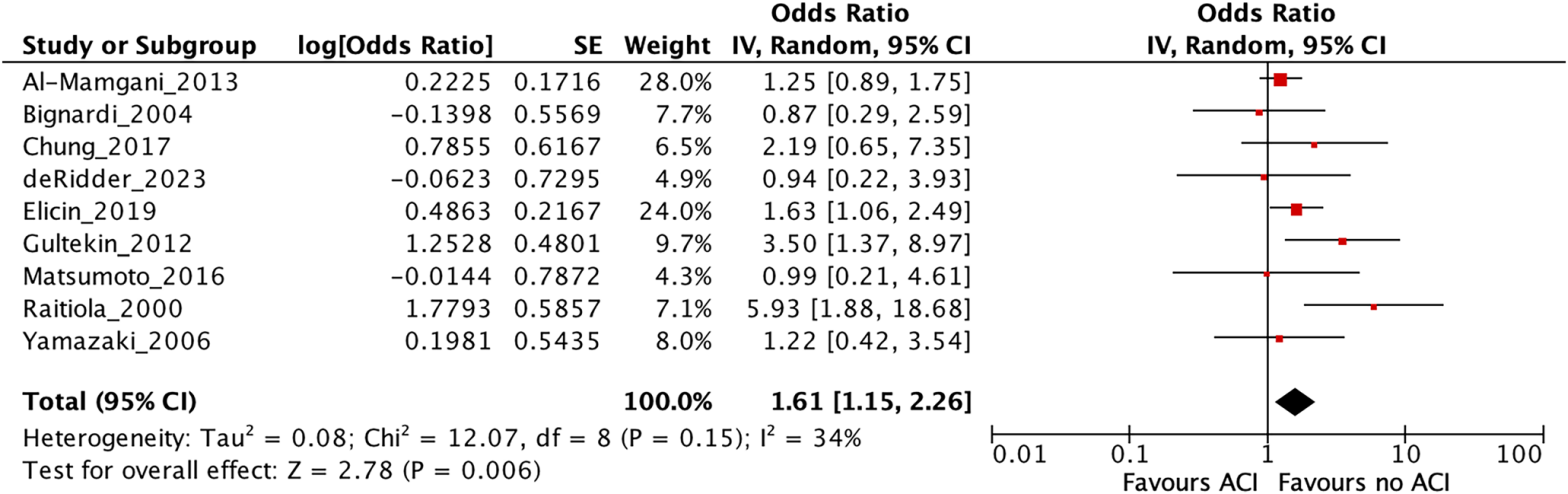
Forest plot. [Color figure can be viewed at wileyonlinelibrary.com]

A univariate meta‐analysis was conducted to evaluate whether ACI is associated with an increased risk of local recurrence in early‐stage glottic carcinoma treated with RT. A total of nine studies were included, comparing the local recurrence rates between patients with and without ACI. The pooled analysis demonstrated a statistically significant association with an overall OR of 1.61, 95% CI: 1.15–2.26 (*p* = 0.006), indicating that patients with ACI have 61% higher odds of experiencing local recurrence compared to those without ACI (Figure 2). Moderate between study heterogeneity was observed (*I* ^2^ = 34%) and the test for overall effect was significant (*Z* = 2.78).

### 3.3 Multivariate Meta‐Analysis

To address the limitations of univariate analysis and to better capture the potential correlation among other risk factors, we conducted a Bayesian MVMA‐MF.

Out of 21 initially identified risk factors, 14 were included in the MVMA‐MF model based on being reported in at least two studies (Table 1). Table 2 presents the median overall log ORs with 95% CrIs for each risk factor under the three Bayesian model assumptions (H, M0, U).

**TABLE 1:** The early‐stage glottic carcinoma data containing nine studies with 14 risk factors.

Study	Risk factors													
	ACI	T substage	Vocal cord mobility	Subglottic extension	RT technique	Total RT dose	Fraction size	OTT	Field size	Gender	Hb level	Smoking status	Age	Tumor grading
Elicin et al. (2019)	0.51 (0.16)	0.48 (0.21)			−0.17 (0.25)	0.45 (0.16)				1.25 (0.59)			−0.04 (0.18)	
Gultekin et al. (2012)	0.73 (0.28)	0.64 (0.30)		0.41 (0.35)	0.30 (0.18)					−0.92 (1.27)		0.97 (1.01)	1.21 (0.30)	−0.08 (0.18)
Yamazaki et al. (2006)	−0.20 (0.66)	0.57 (0.73)					−1.17 (0.41)			−0.62 (1.05)	−0.21 (0.45)	0.25 (0.72)	−0.26 (0.44)	
Al‐Mamgani et al. (2013)	0.10 (0.20)					−0.11 (0.11)	0.47 (0.06)			0.18 (0.56)	0.99 (0.16)	1.31 (0.10)	0.00 (0.25)	
Bignardi et al. (2004)	−0.73 (0.47)		−0.07 (0.63)	0.48 (0.75)	0.34 (0.48)	0.04 (0.08)		0.18 (0.20)	0.17 (0.57)				0.00 (0.02)	
Chung et al. (2018)	0.51 (0.40)	0.72 (0.47)								−0.06 (0.74)			0.37 (0.39)	−0.13 (0.45)
Matsumoto et al. (2016)	−1.03 (0.83)	1.0 (0.68)					1.26 (0.58)	1.03 (0.69)		−0.20 (1.14)			0.92 (0.63)	
Raitiola et al. (2000)	1.34 (0.47)	1.48 (0.46)	1.0 (0.64)											
de Ridder et al. (2023)	−0.36 (0.72)	2.08 (0.82)							0.02 (0.03)	−0.01 (1.07)		2.22 (0.73)	−0.02 (0.04)	

*Note*: The effect size is log odds ratio with within‐study standard error in parentheses. The blank entries indicate that the risk factors are unavailable from the corresponding studies.

Abbreviations: ACI: Anterior commissure involvement; Hb: hemoglobin; OTT: overall treatment time; RT: radiotherapy; T: tumor.

**TABLE 2:** The estimated overall log odds ratios (95% CrI) of the 14 risk factors in the early‐stage glottic carcinoma data obtained by Model H (accounting for both between‐ and within‐study correlations), Model M0 (only accounting for between‐study correlations), and Model U (univariate model ignoring both between‐ and within‐study correlations) using the Bayesian method.

Risk factor	*N* of studies	Estimated overall log odds ratio		
		Model H	Model M0	Model U
ACI	8	0.05 (−1.81 to 1.32)	0.36 (−0.50 to 1.19)	0.36 (−0.13 to 0.82)
T substage	6	0.87 (0.18 to 1.62)	0.79 (0.20 to 1.52)	0.82 (0.36 to 1.42)
Vocal cord mobility	2	0.53 (−4.79 to 8.19)	0.31 (−4.44 to 4.99)	0.44 (−6.05 to 6.95)
Subglottic extension	2	0.51 (−3.74 to 5.55)	0.42 (−3.92 to 4.67)	0.43 (−5.57 to 6.42)
RT technique	2	0.17 (−0.81 to 1.35)	0.15 (−1.70 to 2.02)	0.15 (−1.70 to 2.03)
Total RT dose	3	0.05 (−1.88 to 1.88)	0.14 (−1.94 to 2.28)	0.11 (−1.73 to 1.97)
Fraction size	3	0.08 (−3.99 to 3.53)	0.42 (−3.68 to 4.64)	0.18 (−4.16 to 4.52)
OTT	2	0.59 (−4.14 to 5.13)	0.50 (−4.15 to 5.30)	0.53 (−5.59 to 6.83)
Field size	2	0.15 (−4.62 to 5.57)	0.09 (−4.88 to 5.21)	0.07 (−5.70 to 5.80)
Gender	6	0.00 (−1.76 to 1.32)	0.18 (−1.00 to 1.24)	0.16 (−0.77 to 0.97)
Hb level	2	0.65 (−4.32 to 5.18)	0.70 (−5.10 to 6.42)	0.43 (−6.33 to 7.11)
Smoking status	3	1.07 (−1.13 to 2.82)	1.26 (−0.71 to 3.20)	1.23 (−0.52 to 2.92)
Age	7	0.11 (−1.26 to 0.91)	0.20 (−0.39 to 0.95)	0.15 (−0.17 to 0.59)
Tumor grading	2	−0.14 (−2.66 to 2.31)	−0.11 (−4.97 to 4.70)	−0.10 (−5.77 to 5.49)

Abbreviations: ACI: Anterior commissure involvement; Hb: hemoglobin; N: number; OTT: overall treatment time; RT: radiotherapy; T: tumor.

T substage was the only variable with consistently positive and statistically significant associations across all models (Model H: 0.87, 95% CrIs 0.18–1.62; Model M0: 0.79, 95% CrIs 0.20–1.52; Model U: 0.82, 95% CrIs 0.36–1.42). ACI demonstrated positive but non‐significant associations across all models with 95% CrIs including zero, indicating substantial uncertainty in the estimated effect. Smoking status also showed a trend for increased risk, but all CrIs included zero, reflecting insufficient evidence for a definitive association. For the remaining 11 risk factors, the estimated log ORs were generally small or inconsistent, with wide 95% CrIs crossing zero.

**FIGURE 3:**
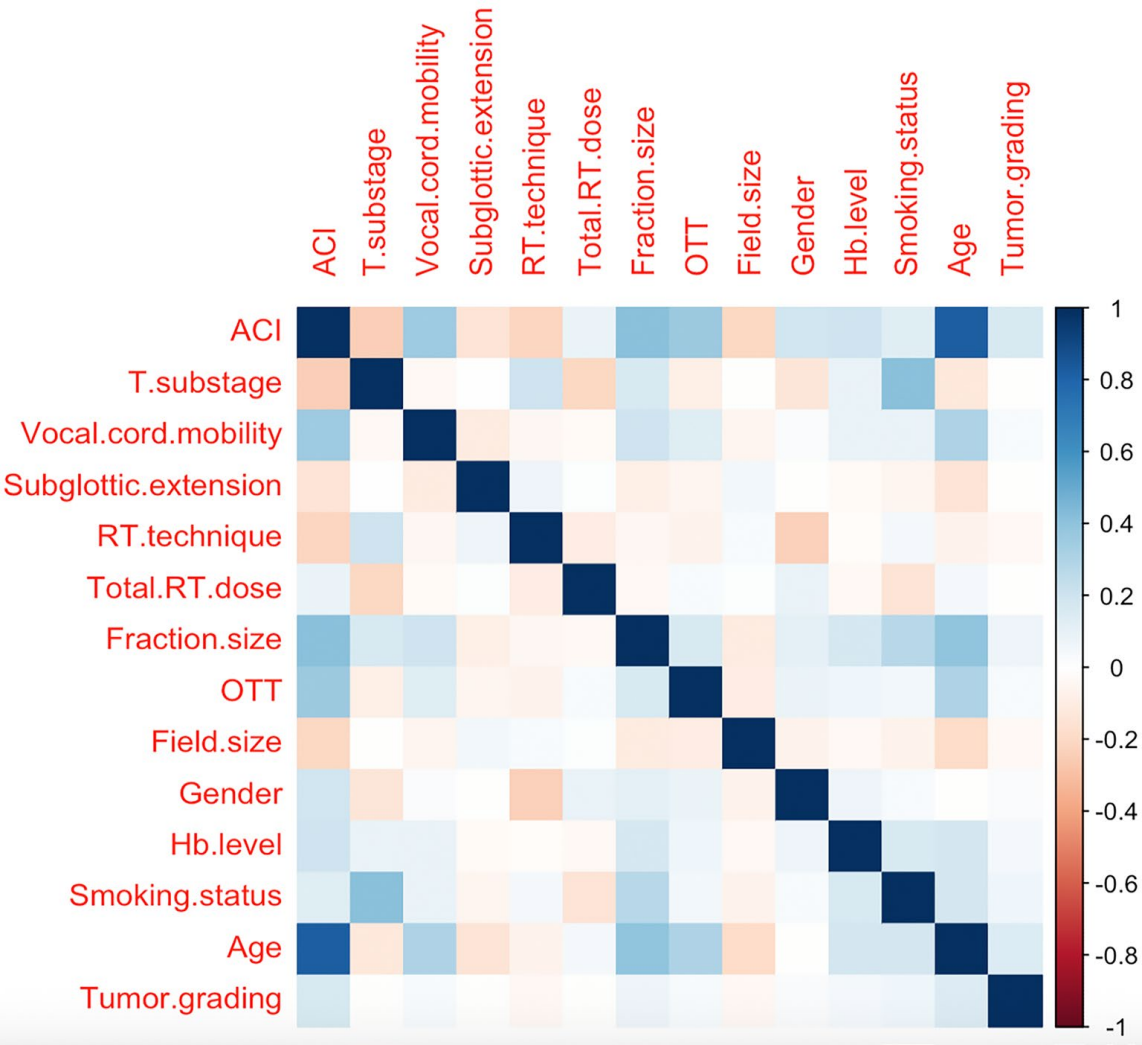
Correlation plot. Plot of the estimated overall correlations between the 14 risk factors produced by Model H in the early‐stage glottic carcinoma data. Darker color implies higher correlation. [Color figure can be viewed at wileyonlinelibrary.com]

Figure 3 illustrates the estimated correlation matrix of the 14 included variables from Model H. Most pairwise correlations were low to moderate, indicating weak to modest linear associations between variables. This supports the appropriateness of the multivariable modeling approach, allowing for the simultaneous evaluation of multiple interrelated risk factors.

### 3.4 ACI Univariate and Multivariate Meta‐Analysis Comparison

To facilitate direct comparison with univariate estimate for ACI (OR: 1.61, 95% CI: 1.15–2.26), the multivariate log ORs for ACI were exponentiated to obtain adjusted ORs. The resulting adjusted ORs for ACI were 1.05 (95% CI: 0.16–3.75) in Model H, 1.43 (95% CI: 0.61–3.29) in Model M0 and 1.43 (95% CI: 0.88–2.27) in Model U.

## 4 Discussion

This study represents the first attempt to apply both univariate and multivariate meta‐analysis to investigate the prognostic role of ACI in early‐stage glottic carcinoma treated with definitive RT.

In the univariate meta‐analysis, ACI was significantly associated with a higher risk of local recurrence (OR 1.61, *p* = 0.006), consistent with the hypothesis that ACI may facilitate tumor spread, potentially limiting local control with RT alone. However, the moderate heterogeneity (*I* ^2^ = 34%) and methodological limitations—particularly the lack of time‐to‐event data (the use of ORs, rather than hazard ratios, does not capture differences in recurrence timing which is clinically relevant)—may compromise the robustness of this finding. To address these limitations and given the potential multicollinearity between ACI and other clinical variables, we applied a Bayesian MVMA‐MF with missing data handling. MVMA‐MF approach is well‐suited for oncological datasets where risk factors are often correlated and incompletely reported. Unlike the univariate meta‐analysis, ACI was not significantly associated with local recurrence in MVMA‐MF, with adjusted ORs reduced to 1.05 (Model H) and 1.43 (Model M0 and U). This discrepancy between univariate and multivariate results highlights the risk of overestimating associations in univariate analysis when confounding or correlated variables are not accounted for. In this context, ACI may be correlated with other prognostic factors, such as T substage, resulting in confounding in the univariate setting. Interestingly, among the 14 risk factors evaluated, T substage emerged as the most consistently and statistically significant predictor of local recurrence across all models. This result reinforces T stage known prognostic value and supports its use as primary stratification variable in clinical decision‐making. Smoking status also showed a positive trend across models, but the lack of statistical significance may reflect insufficient data, inter‐study variability or residual confounding. Most other clinical and RT‐related variables (such as age, total RT dose, OTT, field size) showed weak or inconsistent associations, possibly due to reporting and low statistical power.

Previous univariate meta‐analyses have attempted to recognize the prognostic power of ACI features in early‐stage glottic carcinoma [2, 16]. Both demonstrated an unfavorable outcome in patients with ACI who underwent RT but were limited by moderate to high interstudy heterogeneity. The effect of ACI on local control was examined across 16 studies and both reported a statistically significant difference in favor of patients without ACI (relative risk: 0.904, *p* < 0.001 [2]; absolute difference in local control: 12%, *p* < 0.0001 [16]) with interstudy heterogeneity values of *I* ^2^ 51.7% (high) and 34.8% (moderate), respectively. Our MVMA‐MF was specifically designed to overcome these two major limitations of prior meta‐analyses: heterogeneity and multicollinearity. To reduce the potential heterogeneity, during the screening process, we excluded all studies involving multimodal treatment or patients treated by transoral laser microsurgery (TLM), even if previous evidence does not indicate a statistically significant difference in local control rates between RT and TLM [17]. Furthermore, our dataset focused on patients treated in the 2000s, reflecting outcomes in the era of modern RT, whereas much of the earlier evidence was derived from two‐dimensional RT. To mitigate multicollinearity issue, our analysis accounts for multiple covariates simultaneously, enhancing robustness of the estimated effects.

Therefore, while ACI was a strongly significant factor in univariate analysis, in MVMA‐MF ACI was not significant for further consideration. Cancer staging is an important component of patient care. Although subcategorization of cT1 had been advocated by several studies [18, 19], our data suggested that the addition of ACI criterion is marginal in the distinction between subgroups. Advances in diagnostic imaging, particularly high‐resolution MRI and high‐resolution (4K) laryngoscopy with narrow band imaging (NBI) technology, have greatly improved assessment of local extension in recent decades [20]. These advances underscore the need for updated RT series aligned with contemporary diagnostic and therapeutic approaches, as the choice of high‐resolution imaging modality at diagnosis substantially affects staging accuracy.

Despite the methodological rigor of the MVMA‐MF approach, several limitations should be acknowledged. First, this analysis was based on a limited number of small, single‐institution series. Differences in the variables analyzed and in the availability of univariate results across the screened studies further restricted the inclusion criteria. As a results, only nine studies were included in our final analysis. Large‐scale prospective trials are lacking, and there is no randomized controlled evidence directly comparing early‐stage glottic cancer with or without ACI. Therefore, our results require validation, but because the data largely reflect patients treated in the 2000s, the proposed conclusion would be widely applicable. Second, there was treatment heterogeneity pertaining to the use of different RT technique and dose/fraction among the institutions. To partially address this, RT details were included as covariates in the MVMA‐MF. Third, although 14 prognostic parameters were analyzed, only 4 were available in more than 75% (*n* = 7) of included studies. Many potentially relevant series predated the 2000s, and their original datasets were unavailable despite our attempts to contact authors. Future pragmatic data collection is needed to improve prognostication for early‐stage glottic carcinoma. At present, ACI does not emerge as a fundamental prognostic factor across major statistical aspects, including collinearity, outcome prediction accuracy and weighted distribution. Therefore, the presence of ACI should not be viewed as an automatic indicator of poor RT response or a mandate for surgical resection. ACI should be interpreted in the context of T substage and other clinical variables.

This study demonstrates the value of MVMA‐MF, particularly in contexts where traditional univariate meta‐analysis may be biased or misleading. By accounting for heterogeneity, missing data and correlated variables, this approach provides a more comprehensive and accurate understanding of recurrence risk profile in early‐stage glottic carcinoma treated with definitive RT, supporting the importance of advanced meta‐analytic techniques in complex clinical datasets.

In conclusion, this study presents the first application of Bayesian MVMA‐MF to assess the prognostic role of ACI in predicting local treatment failure in early‐stage glottic carcinoma treated with definitive RT. While univariate analysis suggests that ACI may be associated with increased recurrence risk, MVMA‐FM reveals that this associations is not statistically robust when accounting for correlated risk factors. T substage remains the most consistent predictor of outcome. Overall, this study provides the strongest level of evidence to date that ACI has no independent prognostic value in early‐stage glottic carcinoma.

## Author Contributions

G.S. conceived the study. G.S. and F.D.F. obtained the raw data; all authors had access to datasets and contributed to the creation of labels and definitions. L.P. developed the script and carried out statistical analysis. F.D.F. and G.S. carried out final collation of results. F.D.F. wrote the first draft of the manuscript. G.S. and L.P. contributed to editing and revision. All authors have read and approved the final manuscript.

## Funding

The authors have nothing to report.

## Conflicts of Interest

The authors declare no conflicts of interest.

## Data Availability

The data that support the findings of this study are available on request from the corresponding author. The data are not publicly available due to privacy or ethical restrictions.
